# Genome-Wide Identification and Expression Analysis of *TUA* and *TUB* Genes in Wheat (*Triticum aestivum* L.) during Its Development

**DOI:** 10.3390/plants11243495

**Published:** 2022-12-13

**Authors:** Yang Ren, Qilu Song, Sicong Shan, Junwei Wang, Shoucai Ma, Yulong Song, Lingjian Ma, Gaisheng Zhang, Na Niu

**Affiliations:** 1 Key Laboratory of Crop Heterosis of Shaanxi Province, Wheat Breeding Engineering Research Center, Ministry of Education, College of Agronomy, Northwest A&F University, Yangling 712100, China; 2 College of Plant Protection, Northwest A&F University, Yangling 712100, China

**Keywords:** wheat, tubulin, expression pattern, male sterility

## Abstract

Microtubules play a fundamental role in plant development, morphogenesis, and cytokinesis; they are assembled from heterodimers containing an α-tubulin (TUA) and a β-tubulin (TUB) protein. However, little research has been conducted on the TUA and TUB gene families in hexaploid wheat (*Triticum aestivum* L.). In this study, we identified 15 *TaTUA* and 28 *TaTUB* genes in wheat. Phylogenetic analysis showed that 15 *TaTUA* genes were divided into two major subfamilies, and 28 *TaTUB* genes were divided into five major subfamilies. Mostly, there were similar motif compositions and exon-intron structures among the same subfamilies. Segmental duplication of genes (WGD/segmental) is the main process of TaTUA and TaTUB gene family expansion in wheat. It was found that *TaTUA* and *TaTUB* genes presented specific temporal and spatial characteristics based on the expression profiles of 17 tissues during wheat development using publicly available RNA-seq data. It was worth noting, via qRT-PCR, that two *TaTUA* and five *TaTUB* genes were highly expressed in fertile anthers compared to male sterility. These were quite different between physiological male sterile lines and S-type cytoplasmic male sterile lines at different stages of pollen development. This study offers fundamental information on the TUA and TUB gene families during wheat development and provides new insights for exploring the molecular mechanism of wheat male sterility.

## 1. Introduction

Microtubules are important elements of the cytoskeleton and are found in all eukaryotes. They play an important role in the process of intracellular transport, cell morphology, cell wall deposition, organelle localization, cell motility, signal transduction, cell polarity, and cell division. In vivo, microtubules are highly dynamic, taking part in depolymerization, severing, bundling and nucleation, and transport. These processes are precisely regulated by hundreds of microtubule-associated proteins (MAPs). Microtubules are tubular structures composed of α/β-tubulin heterodimers. α-tubulin (TUA) is composed of 450 amino acid residues and has a molecular mass of 50 kDa. β-Tubulin (TUB) is composed of 455 amino acid residues and about 50 kDa in mass.

In eukaryotes, TUA sequences have 89~95% similarity, and TUB sequences have 88~94% similarity [1]. Different tubulins also have structural differences in net electric charge, dipole moments, and dipole vector orientations [2]. In higher plants, TUA and TUB have many subtypes, and the diversity of these subtypes is owed to two reasons: TUA and TUB are encoded by several different genes, and they are modified after translation [3]. Six genes encode TUA proteins, and nine genes encode TUB proteins in Arabidopsis [4,5]. Furthermore, there are 8 *TUA* genes and 20 *TUB* genes in poplar [6], 4 *TUA* genes and 8 *TUB* genes in rice [7], and 19 *TUB* genes in cotton [8]. The diversity of microtubule subtypes contributes to the assembly of specific microtubule arrays and endows microtubules with different functions, which participate in various biological processes of plant growth and development. Microtubule function is also involved in mitosis and meiosis. In mitosis, tubulins are organized into different microtubule arrays, such as the cortical microtubules, preprophase band (PPB), spindle, and phragmoplast microtubule array, which assist the smooth transition between different stages of cell division. Microtubules cannot form a PPB during meiosis. During microspore division, microtubules are organized into asymmetrical spindles and phragmoplast microtubule arrays, which assist the microspore in dividing twice.

Wheat (*Triticum aestivum* L.) is an economically important food crop worldwide. With improved living standards, the increasing size of the labor force and production costs, and the uncertainty of biotic and abiotic stresses, there is a need to address the challenges of high yields, stable yields, improving quality, reducing costs, and protecting the environment in wheat production. The use of wheat heterosis has been shown to be an effective way to improve both yield and quality. As a self-pollinating crop, it is challenging to create wheat heterosis [9]; male sterility is a much more effective and practical protocol. Although researchers have conducted numerous studies regarding the mechanism, the results are inconclusive.

Our previous studies noted that meiosis and mitosis are abnormal in most types of cytoplasm male sterile (CMS) lines in wheat [10,11,12]. Besides, the failure of microtubules to organize into normal microtubule arrays leads to division failure and the formation of aborted microspores during cell division [13,14,15,16,17]. It is worth studying whether male sterility is caused by abnormal assembly of microtubules. We have little information on the quantity and types of wheat tubulins, which have been shown to be composed of α/β -tubulin heterodimers. Tubulin’s function in wheat growth and development and regulation of cell division is still unclear, so a systematic analysis of the TUA and TUB gene family was performed for the first time in *T.aestivum* L, including gene characteristics, gene structure, cis-acting promoter elements, gene repetition and protein motif, and tubulin gene synteny relationship with other species. We analyzed the expression patterns of *TaTUA* and *TaTUB* genes in different wheat tissues at different developmental stages based on transcriptomic data. Importantly, we compared the expression patterns of two *TaTUA* and five *TaTUB* genes between the cytoplasm of anthers of male sterile lines and fertile wheat lines. The results provided a preliminary reference for further study the functions of *TaTUA* and *TaTUB* genes in wheat growth and development. Furthermore, they provided valuable insights into the mechanism of cytoplasm male sterility in wheat.

## 2. Results

### 2.1. Identification and Physicochemical Characteristics of TUA and TUB Proteins in Wheat

A total of 43 tubulin genes were identified in wheat, including 15 *TaTUA* genes and 28 *TaTUB* genes through HMM analysis, BLASTP alignment, and CDD domain identification (Table 1). Of all genes, 1 *TaTUB* gene mapped to an unclassified chromosome, and the other 42 genes were, respectively, mapped to each chromosome except for 6A and 7B.

All the genes were renamed based on their positions on the chromosomal scaffolds (Table 1). Wheat is a heterologous hexaploidy plant (2n = 6X = BBAADD = 42) with three subgenomes: A, B, and D. Most genes contain redundant homologous copies from subgenomes A, B, and D (called triplets). Blast searches revealed that the 30 genes belonged to the triplets of 10 genes, 4 genes (*TaTUB-6B*, *TaTUB-6D*, *TaTUB-7A*, and *TaTUB-7D*) belonged to twin genes with two homologous copies, and 9 genes had one homolog copy in the subgenomes (Table 1 and Table 2).

The predicted physicochemical properties of the amino acid sequences of TaTUA and TaTUB proteins showed that the 15 *TaTUA* genes encoded proteins containing 449 to 451 amino acids. The MW and pI varied from 49.61 to 49.89 kDa and 4.57 to 4.84, respectively. The subcellular localization predictions suggested that 13 *TaTUA* were located in the cytoplasm and 2 *TaTUA* were located in the Golgi apparatus. The 28 *TaTUB* genes encoded proteins containing 232 to 499 amino acids. The MW and pI varied from 25.06 to 55.81 kDa and 4.4 to 8.68, respectively. The subcellular localization predictions suggested that 15 *TaTUB* were located in the cytoplasm, 6 *TaTUB* were located in the Golgi apparatus (GA), and 7 *TaTUB* were located in plasmids. The GRAVY of TaTUA and TaTUB proteins were negative, indicating that they were hydrophilic.

### 2.2. Gene Duplication and Synteny Analysis of TaTUA and TaTUB

Gene duplications are the primary driving forces in the evolution of genomes and genetics [18]. We explored duplications within the TaTUA and TaTUB gene family using MCScan X. The results indicated that 37 segmental duplication genes (WGD/segmental), 2 tandem duplication genes (tandem), and 4 dispersed duplication genes (dispersed) were discovered in 43 genes (Table 1). The results determined that WGD was the main means of TaTUA and TaTUB gene family expansion in wheat.

**Figure 1:**
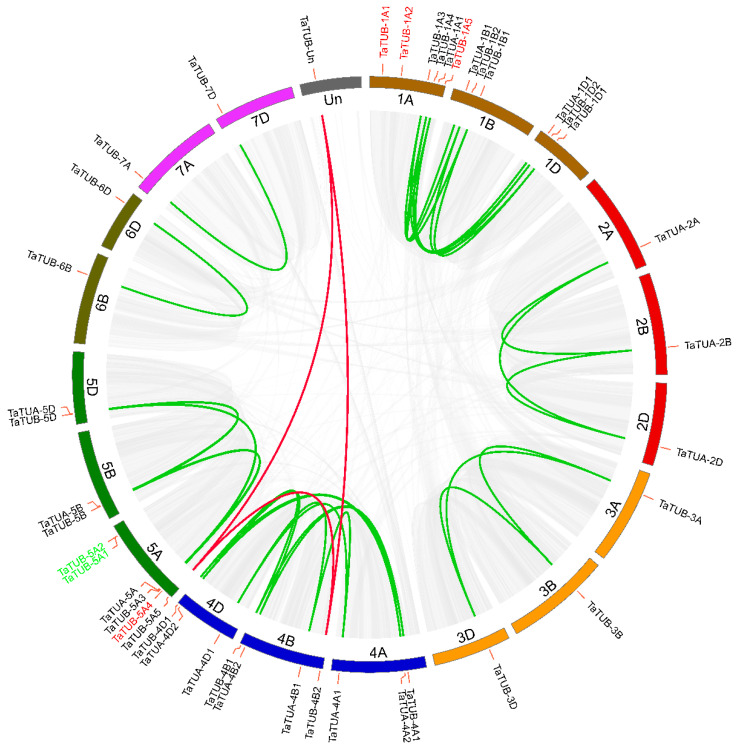
Synteny of the *TaTUA* and *TaTUB* genes in wheat. All syntenic blocks of the wheat genome are linked by grey lines, while colored lines link syntenic relationships of the TaTUA and TaTUB genes.

We performed synteny analysis in wheat to further investigate gene duplications in the TaTUA and TaTUB gene families. The syntenic relationship is shown in Figure 1. The results showed that there were 1719 synteny blocks in the wheat genome, and 78622 synteny gene pairs were found in these synteny blocks. In total, 34 synteny gene pairs had arisen from 37 WGD of *TaTUA* and *TaTUB* genes, and 31 pairs of synteny gene pairs existed between the same group of heterologous chromosomes. For example, *TaTUA-1A1*, *TaTUA-1B1*, and *TaTUA-1D1* were detected in the chromosomes 1A,1B, and 1D, respectively, and three genes formed two synteny gene pairs. *TaTUA-4B2* and *TaTUA-5A5* were located on chromosomes 4B and 5A, respectively, and formed one synteny gene pair. There was a synteny relationship between *TaTUA-4B2, TaTUA-5A5*, and *TaTUB-Un* that was not located on any chromosome.

**Figure 2:**
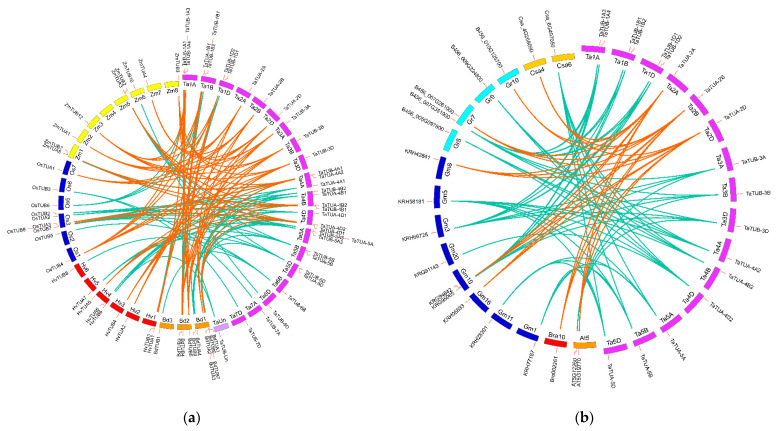
Synteny of *TaTUA* and *TaTUB* genes in wheat with other plant species: (**a**) wheat and four monocot plant species; (**b**) wheat and five dicot plant species. Colored lines link syntenic relationships of the TUA and TUB genes.

To further investigate gene duplications in the TaTUA and TaTUB gene family, we performed synteny analysis between wheat and nine plants to determine the synteny relationship between *TUA* and *TUB* genes of wheat and that of other species. These included four monocot plant species—*Hordeum vulgare*, *Brachypodium distachyon*, *Oryza sativa*, and *Zea mays* (Figure 2A)—and five dicot plant species—*Arabidopsis thaliana*, *Brassica napus*, *Gossypium spp*, *Glycine max*, and *Cucumis sativus* (Figure 2B). The results of synteny analysis between wheat and the four monocot plant species showed that 36 genes had 37, 24, 29, and 31 pairs of synteny gene pairs with 11 *TUA* and *TUB* genes in *B. distachyon*, 9 in *H. vulgare*, 10 in *O. sativa*, and 9 in *Z. mays*, respectively (Figure 2A). Among the 36 genes, 6 *TaTUA* and 7 *TaTUB* genes had synteny relationships with each of the four monocot plant species, which suggested that these 13 genes were present before species differentiation (Figure 2A, yellow lines). Between wheat and the five dicot plant species, a total of 18 *TaTUA* and *TaTUB* genes had 6, 3, 39, 20, and 9 pairs of synteny gene pairs with 2 *TUA* and *TUB* genes in *A. thaliana*, 1 in *B. napus*, 9 in *Gossypium spp*, 4 in *G. max*, and 2 in *C. sativus*, respectively (Figure 2B). Furthermore, 3 of the 18 *TaTUA* and *TaTUB* genes had a synteny relationship with each of the five dicot plant species (Figure 2B, yellow lines).

**Figure 3:**
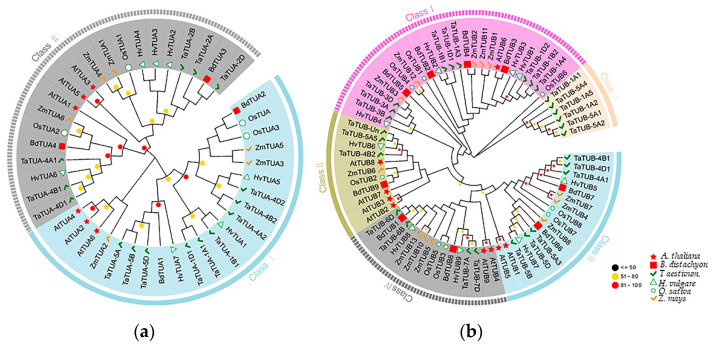
Evolutionary relationships of plant *TUA* and *TUB* genes. (**a**) Evolutionary relationships of *TUA* genes. (**b**) Evolutionary relationships of *TUB* genes. The evolutionary history was inferred using the Neighbor-Joining method. The evolutionary distances were computed using the JTT matrix-based method.

The evolutionary relationship of TaTUA and TaTUB proteins between wheat and five representative plants is shown in Figure 3. A total of 42 *TUA* and 76 *TUB* genes were screened from *A. thaliana*, *H. vulgare*, *B. distachyon*, *O. sativa*, and *Z. mays* using Hmmer searches and BLASTP alignment. TUA were divided into two groups based on their evolutionary relationship, shown in Figure 3A. As shown in the phylogenetic tree of TUA proteins, class I contains nine *TaTUA* genes (*TaTUA-5A*, *5B*, *5D*, *1A1*, *1B1*, *1D1*, *4A2*, *4B2*, and *4D2*), and class II contains six *TaTUA* genes (*TaTUA- 4A1*, *4B1*, *4D1*, *2A*, *2B*, and *2D*). TUB proteins were divided into five groups, as shown in Figure 3B. From the phylogenetic tree of TUB, we found nine *TaTUB*, and most of the *TUB* genes of other monocotyledons were grouped in class I. In contrast, only one *TUB* gene in the dicotyledon Arabidopsis was found in class I. In class II, there were four *TUB* genes in Arabidopsis, three *TaTUB* genes in wheat, and one *TUB* gene each in other monocotyledonous plants, including six *TaTUB* and four *TaTUB* genes grouped in class III and class IV, respectively. Class V only contained six *TUB* genes in wheat.

### 2.3. Gene Structure Analysis of TaTUA and TaTUB

Gene structural diversity and motif type, number, and location are important for the evolutionary and functional analyses of gene family members [19]. The length of most *TaTUA* genes is in the range of 2406 bp to 3658 bp. The length of the *TaTUA-4B1* gene was 5274 bp, significantly different from other *TaTUA* genes. The variation of *TaTUB* gene length was large, ranging from 1430 bp to 6067 bp.

**Figure 4:**
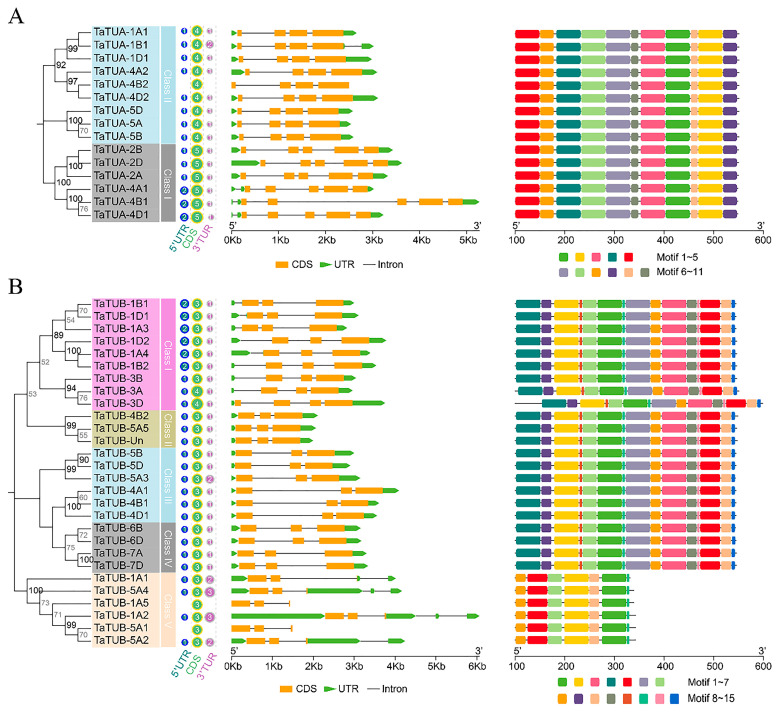
Phylogenetic relationships, gene structure, and architecture of conserved protein motifs in the *TaTUA* (**A**) and *TaTUB* (**B**) genes. The phylogenetic tree was constructed using the maximum likelihood (ML) method. The evolutionary distances of *TaTUA* and *TaTUB* genes were computed using the JTT and WAG matrix-based methods.

Exon-intron analysis is shown in Figure 4. The results showed that three genes (*TaTUA-4B2*, *TaTUB-1A5*, and *TaTUB-5A1*) had no UTR region. Further, 25 genes harbored one 5′UTR and one 3′UTR, 4 genes contained one 5′UTR and two 3′UTR, 2 genes contained one 5′UTR and three 3′UTR, and 9 genes harbored two 5′UTR and one 3′UTR. *TaTUA* in class I and class II contained four and five exons, respectively (Figure 4A). In total, 26 of all 28 *TaTUB* genes contained three exons, and 2 *TaTUB* genes (*TaTUB-3A* and *TaTUB-3D*) had four exons (Figure 4B). Differential exons were located in the 5 ‘UTR region.

Gene structure analysis revealed that most *TaTUA* and *TaTUB* genes in the same group had a similar gene structure, except for members of *TaTUB* genes in class V. The location of exons and the length of introns mainly determined the differences in gene structure among different subclasses. In class II of *TaTUA* genes, *TaTUB-4B1* contained a longer intron, resulting in an extended gene length. The gene structure of *TaTUB* genes in class V was significantly different, mainly caused by differences in the number and length of UTR regions and introns. Overall, 11 conserved motifs of TaTUA proteins and 15 conserved motifs of TaTUB proteins were identified by MEME analysis. All TaTUA proteins contained the same motif, and motif distribution was consistent (Figure 4A). TaTUB proteins had the same motif and distribution patterns in classes I, II, III, and IV, but TaTUB-3D contained an elongated N-terminal. Class V only contained 1, 2, 5, 7, 8, 10, and 13 motifs (Figure 4B).

### 2.4. Promoter Cis-Acting Element Analysis of TaTUA and TaTUB Genes

**Figure 5:**
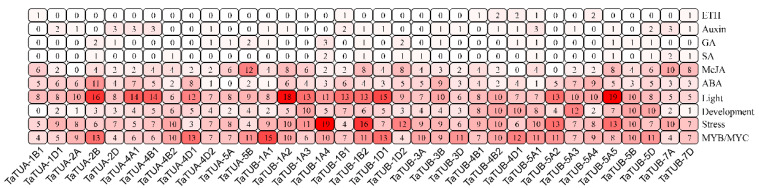
Analysis of the cis-element in *TaTUA* and *TaTUB* gene promoters.

We identified cis-acting elements in the 2000 base pairs upstream of the *TaTUA* and *TaTUB* genes using the PlantCARE database, which is shown in Figure 5. The *TaTUA-1A1* gene was not identified because of its incomplete promoter sequence. The cis-acting elements were divided into 10 categories after removing core promoters, including ethylene response elements (ERE), auxin response elements (TGA-element and AuxRR-core), GA response elements (GARE-motif, P-box, and TATC-box), SA response elements (TCA-element and SARE), MeJA response elements (TGACG-motif and CGTCA-motif), ABA response elements (ABRE), light response elements (G-Box, Sp1, ACE, GT1-motif), developmental-related elements (CCGTCC motif, CAT-box, RY-element, GCN4_motif), stress response-related elements (STRE, ARE, DRE core, GC-motif, WUN-motif), and MYB\MYC transcription factor binding site. Among them, 355 light response elements, 308 MYB\MYC transcription factor binding sites, and 301 stress-related elements accounted for 63.3% of all cis-acting elements. The result suggested that the transcription of *TaTUA* and *TaTUB* genes was regulated by light and the MYB\MYC transcription factor. Meanwhile, they were involved in various biological and abiotic stress response processes. Furthermore, 177 developmental-related elements, 157 ABA, and 147 MeJA response elements were identified, which showed that *TaTUA* and *TaTUB* genes were involved in the development of wheat and were regulated by ABA and MeJA. Cis-acting elements were non-uniform in different gene promoters, indicating that the different genes might have different transcriptional regulation patterns.

### 2.5. Expression Patterns of TaTUA and TaTUB Genes in Different Tissues and Developmental Stages of Wheat

RNA-seq data were used to analyze the expression patterns of all *TaTUA* and *TaTUB* genes according to four types of leaf tissues of nine developmental stages, grains of four developmental stages, the root tissues of six developmental stages, and seven spike tissues of five developmental stages (Figures S1 and S2) [20,21].

*TaTUA* and *TaTUB* genes fell into eight classes in leaf tissues of different developmental stages (Figure S1a). Almost all the genes in class II were highly expressed at all stages. In class III, three TaTUA and nine TaTUB genes were highly expressed at the seedling stage, three-leaf stages, five-leaf stage, and flag leaf stage, while there was a lower expression at the other stages. In classes IV, V, VI, VII, and VIII, only a few *TaTUB* genes were highly expressed.

*TaTUA* and *TaTUB* genes fell into seven classes during grain development (Figure S1b) and fell into six classes in root tissues (Figure S2a). Almost all the genes in classes I and II were highly expressed at each stage in the two tissues. Genes were gradually downregulated with grain development but not in root tissues. Genes in class III were lower expression but showed the same trend. All the *TaTUB* genes in other classes were expressed at lower levels or were not expressed during different developmental stages in two tissues.

*TaTUA* and *TaTUB* genes fell into seven classes in different tissues of the spike at different developmental stages (Figure S2b). Most genes in classes IV and V were expressed at high levels but had a lower expression or no expression in the other classes.

We focused on the expression of genes in anthers. *TaTUA-2A*, *TaTUA-2B*, and *TaTUA-2D* showed higher expression levels, and the genes of *TaTUB-1A4*, *TaTUB-1D2*, *TaTUB-1B2*, and *TaTUB-7A* showed lower expression levels. *TaTUB-3A*, *TaTUB-3B*, *TaTUB-3D*, *TaTUB-1A3*, *TaTUB-1B1*, and *TaTUB-1D1* were notably absent in the anther. Furthermore, *TaTUB-4A1*, *TaTUB-4B1*, and *TaTUB-4D1* were more highly expressed than other genes in the anther.

**Figure 6:**
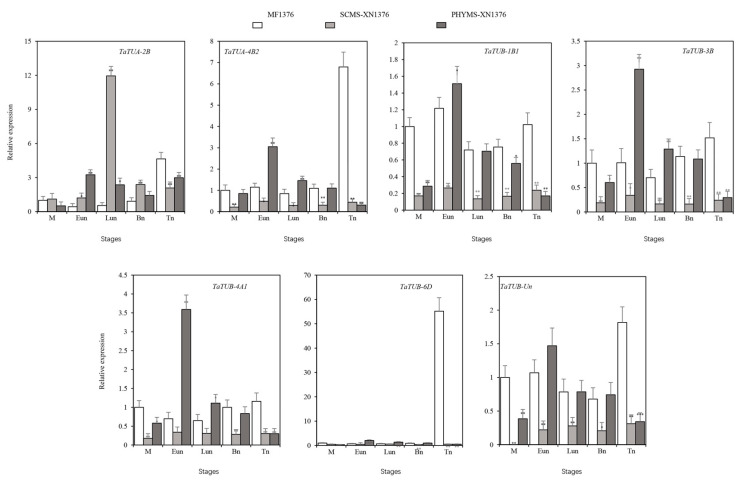
Expression patterns of *TaTUA* and *TaTUB* genes in anthers of MF-XN1376, SCMS-XN1376, and PHYMS-XN1376. MF-XN1376: Xinong 1376; SCMS-XN1376: S-type cytoplasmic male sterile line; PHYMS-XN1376: physiological male sterility; M: Meiosis stage; Eun: Early-uninucleate stage; Lun: Late-uninucleate stage; Bn: Binucleate stage; Tn: Trinucleate stage. An asterisk (*) indicates significance at *p*-values < 0.05, two asterisks (**) indicates significance at *p*-values < 0.01, three asterisks (***) indicates significance at *p*-values < 0.001.

To investigate whether microtubules are involved in the regulation of meiosis and microspore mitosis in wheat pollen mother cells, the anthers of different developmental stages from physiological male sterile lines PHYMS-XN1376, S-types cytoplasmic male sterile lines SCMS-XN1376, and their maintainer lines MF-XN1376 were selected for analysis of the expression patterns of seven *TaTUA* and *TaTUB* genes by qRT-PCR (Figure 6). The expression patterns of seven *TaTUA* and *TaTUB* genes showed significant differences. *TaTUB-6D* showed only slight expression during the stage of anther development. *TaTUA-2B* showed different trends from the other five genes in the first four stages of anther development among three types of materials. Compared to SCMS, the other five genes showed high expression in maintainer lines and PHYMS, except for *TaTUA-2B* at every stage. Five genes showed opposite results between PHYMS-XN1376 and SCMS-XN1376, both at the early and late mononuclear stages. *TaTUA-2B* was upregulated in PHYMS-XN1376 and downregulated in SCMS-XN1376.

## 3. Discussion

Plant TUA and TUB protein sequences are highly conserved, especially in the middle regions of the proteins. These regions control the basic functions of microtubules, being responsible for microtubule polymerization and interactions between dimers [22]. In our study, the analysis of protein domains showed that the different classifications of TaTUA and TaTUB proteins had conserved motifs and were similar in alignment and distribution, except for class V of TaTUB proteins. TaTUB proteins in class V lacked C-terminal sequences, and their molecular weight (MW) was only about 25 kDa. Meanwhile, their distribution of motifs was different from other TaTUB proteins, and the phylogenetic analysis showed no similarity with TaTUB proteins in other species, so these TUB proteins are probably unique to wheat. Although TaTUA and TaTUB proteins were highly conserved, the number and length of introns in different classes of *TaTUA* and *TaTUB* genes were variable. Research shows that introns play an important role in alternative splicing and regulation of gene expression [23,24,25]. Jeon et al. (2000) suggest that tissue-preferential expression of the *OsTuBA1* gene is mediated by intron 1, and it may be involved in a more efficient RNA splicing mechanism [26]. Thus, the intron differences of *TaTUA* and *TaTUB* genes not only enrich the subtypes of TaTUA and TaTUB proteins, but may also be involved in regulating the tissue-preferential expression of genes.

In addition, the number of microtubule genes varies among plant species. For example, there are 4–5 *TUA* genes in *Liriodendron chinense* [27], at least 13 *TUA* genes in cotton [28], 8 *TUB* genes in rice [7], and 20 *TUB* genes in poplars [6]. Furthermore, 15 α-tubulin genes and the chromosome location of 7 α-tubulin genes are identified in hexaploid bread wheat [29]. In this study, 15 *TUA* and 28 *TUB* genes were screened, and all *TUA* and *TUB* genes confirmed their chromosome location; 5 *TUA* genes and 16 *TUB* genes were not homologous in wheat according to ploidy correction. Gene duplications and evolutionary differences among species mainly caused the differences in the number of TUA and TUB genes. Gene duplications and their subsequent divergence play an important role in the evolution of novel gene functions [30]. Based on comparing plants and other eukaryotic genomes, plant genomes tend to evolve at a higher rate, leading to higher genome diversity [31]. WGD/segmental, tandem, transposon-mediated duplication, and retroduplication are the major types of gene duplication [32]. An analysis of the types of gene duplication showed that most *TaTUA* and *TaTUB* genes were part of WGD owing to polyploidization during wheat evolution. Meanwhile, *TaTUB* genes in class V had no synteny relationship with the *TUA* and *TUB* genes in common wheat and ancient wheat species. The evolutionary relationship between these genes and wheat needs to be further studied.

Tubulin is usually expressed at high levels in most tissues because the cytoskeleton requires many microtubules. However, many studies have shown that the expressions of almost all *TUA* and *TUB* genes are controlled both spatially and temporally during plant growth, development, and the process of responding to specific signals. For example, α-tubulin genes play an important role in wheat’s cold acclimation and low-temperature tolerance [33,34]. The different expression patterns of tubulins showed that tubulin subtypes might play specific roles during plant development [22]. In our study, we analyzed the expression patterns of the TaTUA and TaTUB genes in different tissues and developmental stages of wheat. The results showed that not all *TaTUA* and *TaTUB* genes were highly expressed in different wheat tissues, but they did display tissue specificity to some extent. The expression patterns of *TaTUA* and *TaTUB* genes in other tissues and developmental stages of wheat showed that only 13 genes were expressed at high levels in 17 wheat tissues. However, *TaTUA* and *TaTUB* genes were not expressed explicitly in any of the 17 tissues, which suggested that the tissue expression specificity of *TaTUA* and *TaTUB* genes was only relative. Meanwhile, we found that the expression patterns of homologous genes were basically similar, and there was no prominent difference in gene expression levels. The results indicated that *TaTUA* and *TaTUB* homologous genes played a common role in the development of different tissues. Interestingly, *TaTUB* genes in class V showed low or no expression in almost all wheat tissues in this study, which indicated that these genes might be involved in other biological processes rather than the growth and development examined in this study. Furthermore, we further analyzed the different expression patterns of two *TaTUA* and five *TaTUB* genes in anthers of male sterile lines and the maintainer by qRT-PCR; the results showed that they were significantly different in the fertile lines and male sterile lines, and gene expression patterns were also different in anthers of different male sterile wheat types. In particular, *TaTUA-2B* showed different trends from the other five genes in the first four stages of anther development compared to the maintainer, indicating that *TaTUA* and *TaTUB* genes were involved in the regulation of wheat anther development and fertility. However, determining the concrete regulatory function and the mechanism of action still needs further research.

## 4. Materials and Methods

### 4.1. Materials

S-type cytoplasmic male sterile line with the nucleus of common wheat and cytoplasm of *Triticum spelta* L. (named SCMS-XN1376) and its maintainer common wheat Xinong 1376 (called MF-XN1376) were used in this study. The seeds were grown conventionally in the experimental station of Northwest Agriculture and Forestry University, Yangling, China (108° E, 34°150 N). Each row was 1 m long, and the row spacing was 0.25 m. When the wheat developed to Feeks 8.5 (stamens and pistils begin to differentiate), we sprayed SQ-1 to MF-XN1376 at the rate of 5 kg/hm^2^ to achieve physiological male sterility (named PHYMS- XN1376) [35]. PHYMS-XN1376 and MF-XN1376 had the same genotype but different fertility. We collected the anthers of three materials at five developmental stages: meiosis, early mononuclear, late mono-nuclear, binucleate, and trinuclear. The samples were frozen quickly with liquid nitrogen and stored in a refrigerator at −80 °C for future use.

### 4.2. Identification of the Members of the TaTUA and TaTUB Gene Family

The protein, CDS, DNA sequence, and gene annotation file of wheat was downloaded from the Ensembl Plants database (http://plants.ensembl.org/info/data/ftp/index.html, accessed on 1 March 2021). The tubulin family contains a conserved domain called tubulin; the Hidden Markov models (HMMs) of the tubulin domain were downloaded from the Pfam database (http://pfam.xfam.org/, accessed on 10 March 2021). The first search was performed by homologous alignment (*p* < 10^−3^) based on wheat protein sequences. Candidate proteins with *p* < 1 × 10^−26^ were selected to establish new HMM models. The second search and screening of candidate proteins initially obtained members of the wheat tubulin gene family. *TaTUA* and *TaTUB* genes were further screened by homologous alignment, and the candidate tubulin protein sequence was submitted to the SMART (http://smart.embl-heidelberg.de/smart/set_mode.cgi?NORMAL=1, accessed on 12 March 2021) and CDD (https://www.ncbi.nlm.nih.gov/cdd/, accessed on 16 March 2021) databases to confirm the tubulin domain and remove proteins with incomplete domains; all members of TaTUA and TaTUB gene families in wheat were obtained.

### 4.3. TaTUA and TaTUB Gene Structural, Evolution, and Protein Motifs Analysis

The location information of chromosome, exon, intron, and UTR region prediction of the *TaTUA* and *TaTUB* genes were determined from wheat genome annotation information and visualized with GSDS2.0 software [36]. The number of nucleotides, molecular weights (MW), and theoretical isoelectric points (pI) were obtained based on TaTUA and TaTUB protein sequences using the ExPASy database (https://web.expasy.org/compute_pi/, accessed on 19 April 2021). The MEME online database (https://meme-suite.org/meme/, accessed on 2 May 2021) was used to analyze the conserved motifs of TaTUA and TaTUB proteins, with the following parameters: the number of motifs at 15 and visualized with TBtools [37]. All TaTUA and TaTUB proteins were compared in multiple sequences by Clustal X 1.83. The phylogenetic analysis was constructed using MAGA X [37,38].

### 4.4. Promoter Cis-Acting Elements Analysis of TaTUA and TaTUB Genes

The 2000 bp ATG upstream sequence of the *TaTUA* and *TaTUB* genes were extracted from the wheat genome as the promoter region. The promoter region sequence was submitted to the PlantCARE database (http://bioinformatics.psb.ugent.be/webtools/plantcare/html/, accessed on 23 May 2021) for cis-acting element analysis [39], and the main cis-acting elements were visualized using GSDS 2.0 online software [36].

### 4.5. Gene Duplication and Synteny Analysis of TaTUA and TaTUB Genes

Gene duplication types of *TaTUA* and *TaTUB* genes were analyzed using MCScanX. Local BLAST searches were performed based on the whole protein group alignment of TaTUA and TaTUB proteins; synteny gene blocks and pairs were obtained using MCScanX in the wheat genome [40], and the synteny gene pairs were visualized using Circos software [41].

### 4.6. RNA Extraction, Reverse Transcription, and qRT-PCR Analysis

RNA in anthers was extracted according to the instructions in the RNA Extraction Kit (SUM7806, summer BIOTECH, Beijing, China). Total RNA (1 μg) was subjected to gDNA removal followed by reverse transcription reaction, according to the manufacturer’s instructions. The specific primers of qRT-PCR were designed by Oligo 7 software [42], and the specificity and amplification efficiency of the primers were detected by qRT-PCR. *TaActin* was used as a reference gene [43]. Primer sequences are shown in Table 3. The primers were efficient by gel electrophoresis and qRT-PCR (Figures S3 and S4). Actin was used as the reference gene. Relative expression levels were calculated using the 2^−ΔΔCt^ method [44]. Statistical analyses and significant difference tests were analyzed by SPSS Statistics 22. The data and charts were processed using Origin 8.0.

## 5. Conclusions

In this study, a comprehensive analysis demonstrated the importance of *TaTUA* and *TaTUB* genes in wheat development, and 15 *TaTUA* and 28 *TaTUB* genes were identified. We analyzed and established the physicochemical characteristics, prediction of subcellular localization, gene duplication, sequence characterization, chromosome location, and evolutionary relationships. The expression patterns of the *TaTUA* and *TaTUB* genes provide evidence of the potential functions of these genes in different tissues and developmental stages of wheat. Moreover, analysis of *TUB-1B1*, *TUA-2B*, *TUB-3B*, *TUB-4A1*, *TUA-4B2*, *TUB-6D*, and *TUB-Un* gene expression in anthers of male sterile lines showed that *TaTUA* and *TaTUB* genes were involved in the regulation of wheat anther development and fertility. This genome-wide analysis of the TaTUA and TaTUB family will be useful for further exploring the molecular mechanism of TaTUAs and TaTUBs in wheat male sterility.

## Figures and Tables

**Table 1:** Identification of *TaTUA* and *TaTUB* genes, physicochemical characteristics and subcellular localization of TaTUA and TaTUB proteins, and gene duplication patterns.

Gene ID	Gene Name	Chr	Length	MW (kDa)	pI	Gravy	Location	Gene Duplicate Patterns
TraesCS1A02G350200	*TaTUA-1A1*	1A	451	49.71	4.65	−0.20	GA	WGD/segmental
TraesCS1B02G364500	*TaTUA-1B1*	1B	451	49.71	4.65	−0.20	cytosol	WGD/segmental
TraesCS1D02G353100	*TaTUA-1D1*	1D	451	49.71	4.65	−0.20	cytosol	WGD/segmental
TraesCS2A02G185500	*TaTUA-2A*	2A	450	49.61	4.72	−0.15	cytosol	WGD/segmental
TraesCS2B02G221800	*TaTUA-2B*	2B	450	49.61	4.72	−0.15	cytosol	WGD/segmental
TraesCS2D02G202200	*TaTUA-2D*	2D	450	49.61	4.72	−0.15	cytosol	WGD/segmental
TraesCS4A02G065700	*TaTUA-4A1*	4A	449	49.87	4.84	−0.19	cytosol	WGD/segmental
TraesCS4A02G257800	*TaTUA-4A2*	4A	451	49.73	4.65	−0.20	cytosol	WGD/segmental
TraesCS4B02G056700	*TaTUA-4B2*	4B	451	49.73	4.65	−0.20	cytosol	WGD/segmental
TraesCS4B02G243100	*TaTUA-4B1*	4B	449	49.89	4.84	−0.19	cytosol	WGD/segmental
TraesCS4D02G057000	*TaTUA-4D2*	4D	451	49.73	4.65	−0.20	cytosol	WGD/segmental
TraesCS4D02G242700	*TaTUA-4D1*	4D	449	49.89	4.84	−0.19	cytosol	WGD/segmental
TraesCS5A02G407800	*TaTUA-5A*	5A	451	49.79	4.57	−0.22	GA	WGD/segmental
TraesCS5B02G412700	*TaTUA-5B*	5B	451	49.79	4.57	−0.22	cytosol	WGD/segmental
TraesCS5D02G417700	*TaTUA-5D*	5D	451	49.77	4.6	−0.22	cytosol	WGD/segmental
TraesCS1A02G101000	*TaTUB-1A1*	1A	232	25.06	7.31	−0.23	plastid	dispersed
TraesCS1A02G142800	*TaTUB-1A2*	1A	243	26.04	8.68	−0.33	plastid	dispersed
TraesCS1A02G258800	*TaTUB-1A3*	1A	445	50.14	4.48	−0.36	GA	WGD/segmental
TraesCS1A02G309700	*TaTUB-1A4*	1A	447	50.31	4.45	−0.34	GA	WGD/segmental
TraesCS1A02G437700	*TaTUB-1A5*	1A	239	25.69	6.5	−0.33	plastid	dispersed
TraesCS1B02G269400	*TaTUB-1B1*	1B	445	50.15	4.49	−0.36	cytosol	WGD/segmental
TraesCS1B02G320800	*TaTUB-1B2*	1B	447	50.31	4.45	−0.34	cytosol	WGD/segmental
TraesCS1D02G258800	*TaTUB-1D1*	1D	445	50.15	4.49	−0.36	cytosol	WGD/segmental
TraesCS1D02G309200	*TaTUB-1D2*	1D	447	50.31	4.45	−0.34	cytosol	WGD/segmental
TraesCS3A02G333300	*TaTUB-3A*	3A	451	50.68	4.45	−0.36	cytosol	WGD/segmental
TraesCS3B02G363500	*TaTUB-3B*	3B	447	50.21	4.42	−0.37	GA	WGD/segmental
TraesCS3D02G326900	*TaTUB-3D*	3D	499	55.81	4.8	−0.36	plastid	WGD/segmental
TraesCS4A02G296600	*TaTUB-4A1*	4A	445	49.73	4.46	−0.35	cytosol	WGD/segmental
TraesCS4B02G017100	*TaTUB-4B1*	4B	445	49.76	4.47	−0.36	cytosol	WGD/segmental
TraesCS4B02G366500	*TaTUB-4B2*	4B	449	50.24	4.41	−0.34	cytosol	WGD/segmental
TraesCS4D02G015400	*TaTUB-4D1*	4D	445	49.76	4.47	−0.35	cytosol	WGD/segmental
TraesCS5A02G069700	*TaTUB-5A1*	5A	243	26.01	8.68	−0.30	plastid	tandem
TraesCS5A02G069800	*TaTUB-5A2*	5A	243	26.02	8.49	−0.28	plastid	tandem
TraesCS5A02G416400	*TaTUB-5A3*	5A	444	49.84	4.53	−0.36	cytosol	WGD/segmental
TraesCS5A02G443500	*TaTUB-5A4*	5A	239	25.53	6.88	−0.34	plastid	dispersed
TraesCS5A02G534300	*TaTUB-5A5*	5A	447	50.10	4.45	−0.35	cytosol	WGD/segmental
TraesCS5B02G418700	*TaTUB-5B*	5B	445	49.97	4.5	−0.37	GA	WGD/segmental
TraesCS5D02G424100	*TaTUB-5D*	5D	445	49.97	4.5	−0.37	cytosol	WGD/segmental
TraesCS6B02G169300	*TaTUB-6B*	6B	444	49.87	4.4	−0.38	cytosol	WGD/segmental
TraesCS6D02G130500	*TaTUB-6D*	6D	445	49.96	4.4	−0.37	GA	WGD/segmental
TraesCS7A02G466600	*TaTUB-7A*	7A	446	50.21	4.43	−0.39	GA	WGD/segmental
TraesCS7D02G454200	*TaTUB-7D*	7D	446	50.21	4.43	−0.39	cytosol	WGD/segmental
TraesCSU02G137900	*TaTUB-Un*	Un	448	50.21	4.45	−0.34	cytosol	WGD/segmental

**Table 2:** The subgenome distribution of TaTUA and TaTUB homeologs.

Number of Homologous Loci	Distribution of Genome	Number of Genes	Total Number of Genes
Three homeologs	A, B, D	10	30
Two homeologs	A, D	1	2
	B, D	1	2
One homeolog	A	7	14
	B	1	2
	Un	1	2
Total			43

**Table 3:** Primers in this study.

Gene	Forward Primer	Reverse Primer
*TaActin*	ACCTTCAGTTGCCCAGCAAT	CAGAGTCGAGCACAATACCAGTTG
*TUB-1B1*	AGCACCAAGGAGGTTGATGAACA	GTTGCCGATGAAGGTGGACG
*TUA-2B*	AGCTCATCTCTGGGAAGGAGG	GATCCAGTTCCACCACCAACA
*TUB-3B*	ACAAAGGAGGTGGACGAGCAG	TGGAGGTCGAGTTGCCAACAA
*TUB-4A1*	GACGCCAAGAACATGATGTGTG	TCTGCTCATCCACCTCCTTTGT
*TUA-4B2*	TTTGTTGATCTTGAGCCCACTGT	TGATACGGTCCAGGCATAGGTC
*TUB-6D*	GTCAGCTGAACTCCGACCTC	GGGTCAACTCAGGAACAGTGA
*TUB-Un*	CTCCACCTTCATCGGCAACTC	GGCCTCGGTGAACTCCATC

## Data Availability

The data presented in this study are available in the article.

## Supplementary Materials

The following supporting information can be downloaded at: https://www.mdpi.com/article/10.3390/plants11243495/s1. Figure S1: Expression patterns of TaTUA and TaTUB genes in wheat leaf and grain during various development stages; Figure S2: Expression patterns of TaTUA and TaTUB genes in wheat root and different tissues of the spike during various developmental stages; Figure S3: The gel electrophoresis of the primers; Figure S4: The standard curves of the primers.
