# Supplementary material for: Genome-Wide Identification and Expression Analysis of TUA and TUB Genes in Wheat (Triticum aestivum L.) during Its Development
Source: Plants (Basel). 2022 Dec 13;11(24):3495. doi: 10.3390/plants11243495 (PMC9785050; doi:10.3390/plants11243495)
Supplement: Supplementary file 1 [file plants-11-03495-s001.zip › plants-2054671-supplementary.pdf]

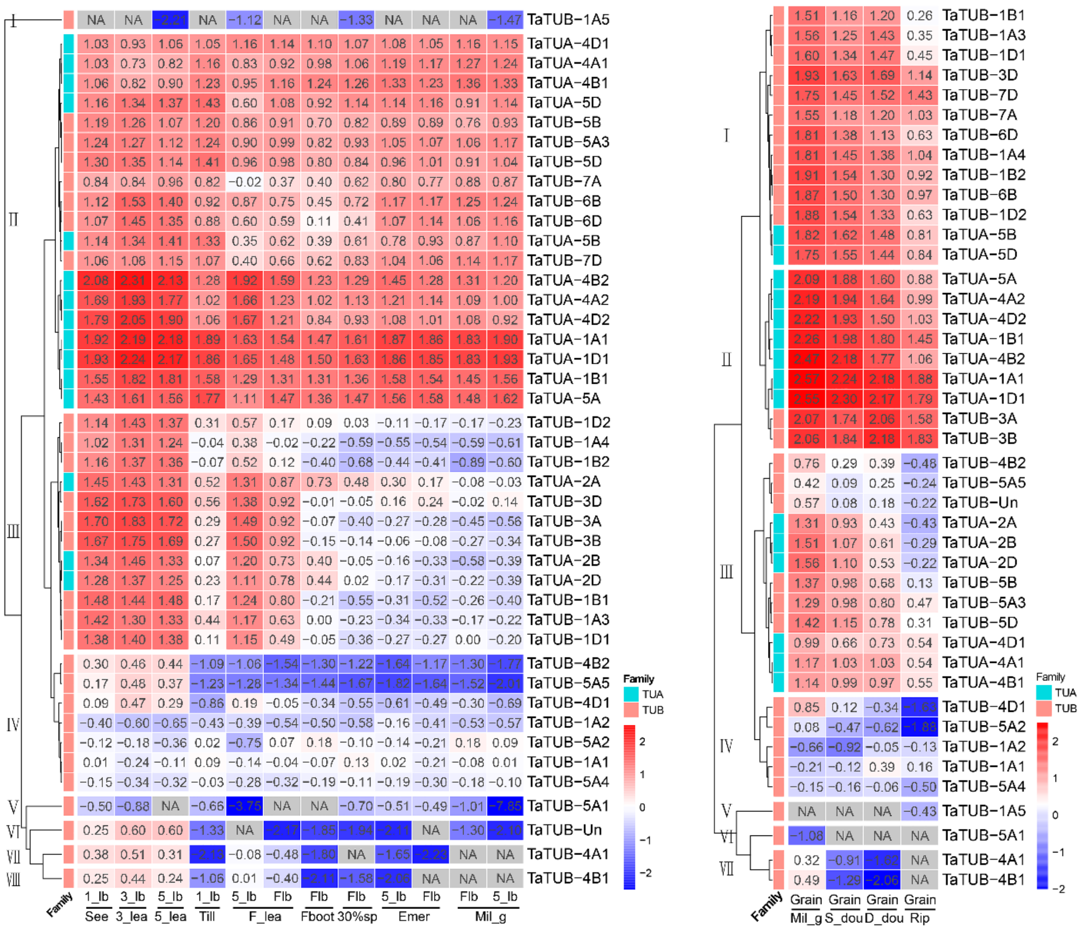

(a) (b)

Figure S1. Expression patterns of *TaTUA* and *TaTUB* genes in wheat leaf and grain during various development stages. (**a**) Expression patterns of *TaTUA* and *TaTUB* genes in wheat leaf; (**b**) Expression patterns of *TaTUA* and *TaTUB* genes in grain. Abbreviations above the line represent organs (tissues) and abbreviations below the line represent developmental stages. 1_lb: first leaf blade; 3_lb: third leaf blade; 5_lb: fifth leaf blade; flb: flag leaf blade; Grain: grain; See: seeding stage; 3_lea: three leaf stage; 5_lea: fifth leaf stage; Till: tillering stage; F_lea: flag leaf stage; Fboot: full boot; 30%sp: 30% spike; Emer: ear emergence; Mil_g: milk grain stage; S_dou: soft dough; D_dou: hard dough; Rip: ripe stage.

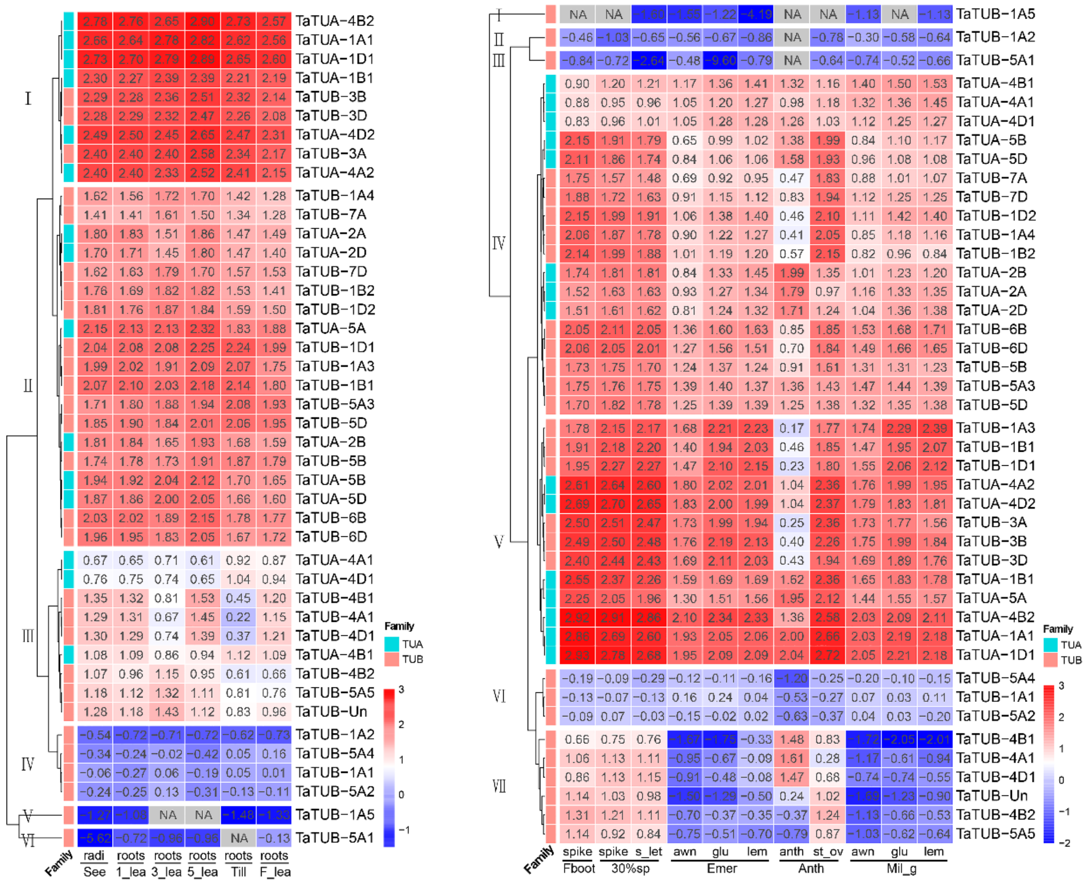

(a) (b)

Figure S2. Expression patterns of *TaTUA* and *TaTUB* genes in wheat root and different tissues of the spike during various developmental stages. (**a**) Expression patterns of *TaTUA* and *TaTUB* genes in wheat root; (**b**) Expression patterns of *TaTUA* and *TaTUB* genes in different tissues of the spike. Abbreviations above the line represent organs (tissues) and abbreviations below the line represent developmental stages. radi: radicle; roots: roots; spike: spike; s_let: spike-lets; awn: awns; glu: glumes; lem: lemma; anth: anther; st_ov: stigma and ovary; See: seeding stage; 1_lea: first leaf stage 3_lea: three leaf stage; 5_lea: fifth leaf stage; Till: tillering stage; F_lea: flag leaf stage; Fboot: full boot; 30%sp: 30% spike; Emer: ear emergence; Anth: anthesis; Mil_g: milk grain stage.

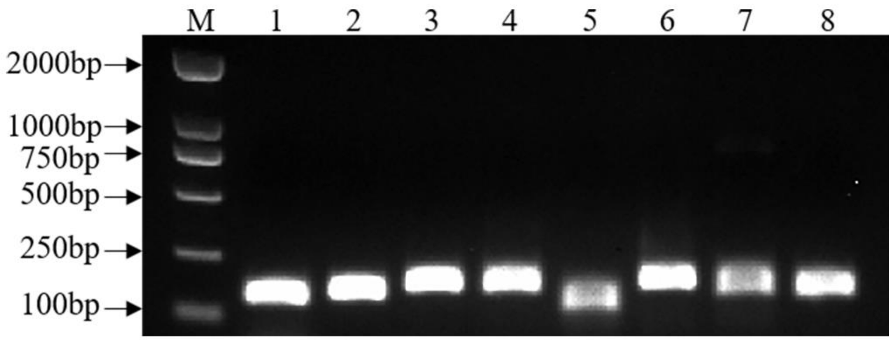

Figure S3. The gel electrophoresis of the primers. M: marker; 1: *TaActin*; 2: *TUB-1B1*; 3: *TUA-2B*; 4: *TUB-3B*; 5: *TUB-4A1*; 6: *TUA-4B2*; 7: *TUB-6D*; 8: *TUB-Un*.

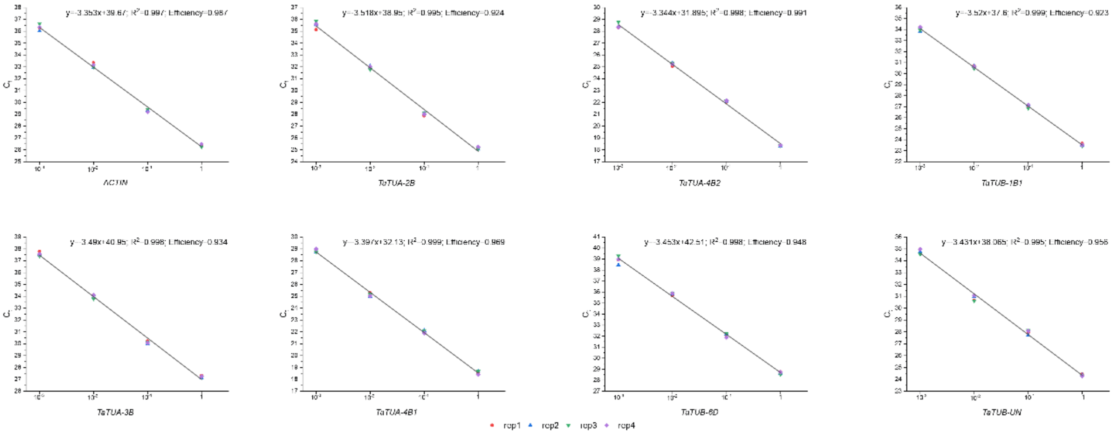

Figure S4. The standard curves of the primers.
